# Differentially expressed genes and key molecules of *BRCA1/2*-mutant breast cancer: evidence from bioinformatics analyses

**DOI:** 10.7717/peerj.8403

**Published:** 2020-01-21

**Authors:** Yue Li, Xiaoyan Zhou, Jiali Liu, Yang Yin, Xiaohong Yuan, Ruihua Yang, Qi Wang, Jing Ji, Qian He

**Affiliations:** 1 Department of Clinical Laboratories, Second Affiliated Hospital of Xi’an Jiaotong University, Xi’an, China; 2 Department of Clinical Laboratories, XIAN XD Group Hospital, Xi’an, China

**Keywords:** Breast cancer, BRCA1/2 mutations, Differentially expressed genes, Survival analysis, diagnostic value

## Abstract

**Background:**

*BRCA1* and *BRCA2* genes are currently proven to be closely related to high lifetime risks of breast cancer. To date, the closely related genes to *BRCA1/2* mutations in breast cancer remains to be fully elucidated. This study aims to identify the gene expression profiles and interaction networks influenced by *BRCA1/2* mutations, so as to reflect underlying disease mechanisms and provide new biomarkers for breast cancer diagnosis or prognosis.

**Methods:**

Gene expression profiles from The Cancer Genome Atlas (TCGA) database were downloaded and combined with cBioPortal website to identify exact breast cancer patients with *BRCA1/2* mutations. Gene set enrichment analysis (GSEA) was used to analyze some enriched pathways and biological processes associated *BRCA* mutations. For *BRCA1/2*-mutant breast cancer, wild-type breast cancer and corresponding normal tissues, three independent differentially expressed genes (DEGs) analysis were performed to validate potential hub genes with each other. Protein–protein interaction (PPI) networks, survival analysis and diagnostic value assessment helped identify key genes associated with *BRCA1/2* mutations.

**Results:**

The regulation process of cell cycle was significantly enriched in mutant group compared with wild-type group. A total of 294 genes were identified after analysis of DEGs between mutant patients and wild-type patients. Interestingly, by the other two comparisons, we identified 43 overlapping genes that not only significantly expressed in wild-type breast cancer patients relative to normal tissues, but more significantly expressed in *BRCA1/2*-mutant breast patients. Based on the STRING database and cytoscape software, we constructed a PPI network using 294 DEGs. Through topological analysis scores of the PPI network and 43 overlapping genes, we sought to select some genes, thereby using survival analysis and diagnostic value assessment to identify key genes pertaining to *BRCA1/2*-mutant breast cancer. *CCNE1*, *NPBWR1*, *A2ML1*, *EXO1* and *TTK* displayed good prognostic/diagnostic value for breast cancer and *BRCA1/2*-mutant breast cancer.

**Conclusion:**

Our research provides comprehensive and new insights for the identification of biomarkers connected with *BRCA* mutations, availing diagnosis and treatment of breast cancer and *BRCA1/2*-mutant breast cancer patients.

## Introduction

Breast cancer susceptibility gene (*BRCA1* and *BRCA2*) mutations, which confer substantial lifetime risks of breast and ovarian cancers (Atchley et al., 2008), influence oncogenesis and metastasis of breast cancer (Kuchenbaecker et al., 2017). *BRCA1/2* are currently proven to be closely related to hereditary breast cancer and some sporadic breast cancer. But there is a paucity of data pertaining to ethnical high-risk cases with *BRCA1/2* mutations and further large *BRCA* mutation prevalence studies (Bernstein-Molho et al., 2019; Armstrong et al., 2019). Although some genes have been identified and the pathogenic mechanism of *BRCA1/2* genes for breast cancer has partly explained, the closely related genes to *BRCA1/2* in breast cancer (BC) remain to be fully elucidated.

The identification of *BRCA1/2* mutation carriers only relies on, genetic testing for high-risk patients judged by their information that have family history or initial clinical symptoms (Foulkes, 2013; Shimada et al., 2019). In fact, this also limits the opportunity of prevention for *BRCA1/2*-mutant breast cancer and other tumors such as ovarian cancer, due to the cost effectiveness for extending to population-based sequencing (sequencing costs not offset by healthcare benefits of preventing future malignancies) (Gourley, 2019) and limitations of *BRCA* gene mutation detection. Loss of one copy of functional BRCA1/2 is not clinically apparent, and somatic mutations detection of BRCA genes is affected by cancer cell content and mutation ratio, lacking the accuracy and inherent simplicity, and the accuracy of detection. Although germline and somatic variants of BRCA1/2 have been described, variants in their genetic regions only account for a small proportion of cancer risk, and the majority is currently unknown, which remains a difficulty for genetic testing (Santana Dos Santos et al., 2018). Moreover, BRCA1/2 mutations render tumors more sensitive to drugs that cause DNA cross-linking, such as cisplatin, carboplatin, and mitomycin. In clinical practices, PARP1 inhibitors, represented by olaparib, have become monotherapies for patients with *BRCA*-mutant cancer (Tutuncuoglu & Krogan, 2019), but perhaps inevitably, long-term effectiveness of which is hindered by their progressive resistance (Barber et al., 2013; Macedo & Ashton-Prolla, 2019). Due to the difficulty in identifying and treating *BRCA1/2*-mutant BC, it is of great importance to find more key candidate genes for the diagnosis and treatment of BC, especially for some hereditary and sporadic BC, and understand underlying pathogenesis mechanisms of *BRCA* mutations.

In recent years, large-scale genome sequencing, such as high-throughput data including The Cancer Genome Atlas (TCGA) database, provides a new method to help researchers explore the complex relationship between genetic molecules and disease (Huang & Li, 2017; Zhai et al., 2019). So, in this study, we screened the transcriptome sequencing dataset of appropriate BRCA mutant and wild-type BC patients from the TCGA database, and thereby identified differentially expressed genes (DEGs) through analysis of these two sets of data to reflect gene expression profiles influenced by *BRCA1/2* mutations, combined with Gene set enrichment analysis (GSEA), survival analysis and diagnostic value assessment. Protein–protein interaction (PPI), survival analysis and diagnostic value assessment help us identify key genes associated with *BRCA1/2* mutations and provide new insights for the specific mechanisms and treatment targets research of *BRCA*-mutant breast cancer different from other breast cancers.

## Material and Methods

### RNA-seq data

**Figure 1:**
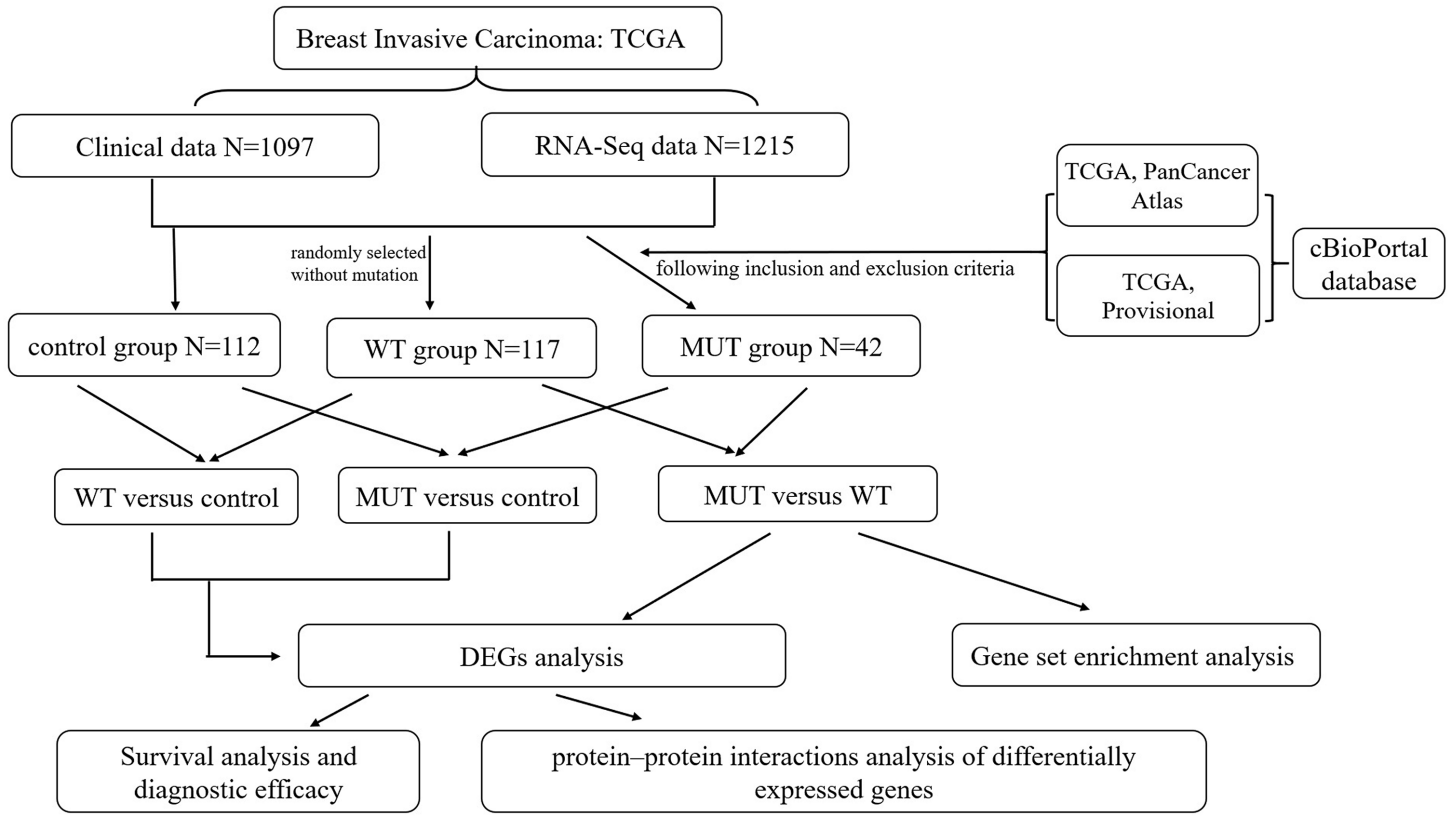
Flow chart of methodologies used in this study. Note: The *BRCA1/2*-mutant (MUT) group was set following the inclusion and exclusion criteria: (1) Data were included when (i) mutation or CNV was shown in both data sets (TCGA PanCancer Atlas and TCGA Provisional) searching by cBioPortal website, (ii) data had complete RNA-seq data and clinical data; (2) Data were directly excluded when amplification was detected in any data set for corresponding samples. The *BRCA1/2* wild-type (WT) group was randomly selected without mutation from all breast cancer RNA-seq data, and had complete RNA-seq data and clinical data. We also classified para-carcinoma samples as control group. And then three differentially expressed genes (DEGs) analysis were performed on three groups in pairs, namely MUT versus (vs.) WT, MUT vs. Control and WT vs. Control. This was followed by applying a PPI integration using the differentially expressed genes in MUT vs. WT as input. The DEGs results of three comparison helped us further screen and identify key candidate genes, and conduct survival analysis and evaluate the diagnostic efficacy for genes closely associated with *BRCA1/2* mutations.

An RNA-Seq dataset of breast cancer, which included the whole transcriptome sequencing dataset and corresponding clinical profiles of over 1000 human BC patients, was download from TCGA database (https://portal.gdc.cancer.gov/). The format of mRNA-seq data is HTseq-Counts which can be analyzed for differential gene expression using the edgeR package, and HTseq-FPKM for functional annotation, pathway enrichment and diagnostic value. The corresponding information related to patients with *BRCA1/2* mutations (MUT) was obtained from the cBioPortal website (http://www.cbioportal.org/index.do) (Gao et al., 2013), including mutation and copy number variation (CNV), in order to create MUT group satisfying *BRCA1* or *BRCA2* mutation with complete RNA-seq data and clinical data. The *BRCA1/2* wild-type (WT) group was randomly selected without mutation from all breast cancer RNA-seq data, and had complete RNA-seq data and clinical data. Moreover, we chose all correspondent para-carcinoma tissue samples from BC RNA-seq data as control group, and the total number is 112. The overall schematic of methods used in this study is shown in Fig. 1.

### Gene set enrichment analysis (GSEA)

To study the effect of *BRCA1/2* mutations on various biological function gene sets in breast cancer patients, GSEA was adopted to analyze the differences of two groups (with or without *BRCA* mutations) in gene mRNA expression levels of biological functional annotation and pathways. Hereby, the number of permutations was set at 1,000, and the remaining were default parameters. Reference gene sets database from Molecular Signatures Database (MSigDB) of h(h.all.v6.2.symbols.gmt), c2 (c2.cp.kegg.v6.2.symbols.gmt) and c5 (c5.bp.v6.2.symbols.gmt; c5.mf.v6.2.symbols.gmt; c5.cc.v6.2.symbols.gmt; consist of genes annotated by the same GO terms), respectively (Liberzon et al., 2015). The MSigDB of h was a hallmark gene sets, constructed on marker genes associated with various cellular biological processes including cell apoptotic and division; c2 was a pathway gene set, which was curated from publications and extracted from canonical pathways and experimental signatures; c5 was Gene Ontology(GO) gene sets, consisted of biological process(BP), cellular component and molecular function. Enrichment analysis was considered statistically significant when meeting the following criterion: nominal *P*-value cutoff (NOM *p*-value) <0.05 and false discovery rate (FDR) <0.25.

### Identification of differential gene expression (DEGs)

Expression profile data of the analyzed groups in the study (MUT vs WT; MUT vs control; WT vs control), were managed by gene ID conversion and default value removal using R package. A total of 19611 genes per sample were available for analysis in the matrix file. EdgeR, an R package for examining DEGs of RNA-Seq count data, was used three times alone without interference, to identify differentially expressed genes between BRCA1/2-mutant (MUT) BC patients and wild-type (WT) BC patients, between MUT and control samples, between WT BC and control samples, respectively. Differentially expressed genes were corrected by FDR adjustment and considered significant following the criterion: —log2 fold change (FC)— ≥1; both the *P*-value and FDR<0.05. We mainly used differentially expressed genes between *BRCA1/2-* mutant BC patients and wild-type BC patients to conduct further analysis, including functional annotation, pathway enrichment and PPI analysis. Moreover, survival analysis and diagnostic efficacy were performed, based on the identification of more meaning genes which were considered differentially expressed in all three comparisons.

### Protein–protein interaction (PPI) network construction

For the DEGs between *BRCA1/2*-mutant BC patients and wild-type BC patients, PPI construction helped our understand relationships of these genetic expression changes, closely related to *BRCA1/2* mutations. We performed PPI network by STRING database (https://string-db.org/), a common online approach known to predict protein-protein interactions, followed by PPI network visualization in Cytoscape (V.3.7.0). Based on the results from the STRING database and analysis from Cytoscape and its plug-in cytohubba, we synthetically evaluated all the genes by 12 topological analysis methods including Degree, Clustering Coefficient and so on, provided by cytohubba (Chin et al., 2014), to identify some specific hub genes closely related to *BRCA* mutations.

### Further analysis

Key candidate genes which were all considered differentially expressed in all three comparisons, were screened to evaluate their prognosis and diagnosis information for breast cancer and *BRCA*-mutant breast cancer. For this purpose, we used Kaplan–Meier plotter (http://kmplot.com/analysis/), a software available online that specializes for survival analysis (Gyorffy et al., 2010). Herein, the overall survivals (OS) of BC patients were analyzed using the Kaplan–Meier method, based on the classification where patients were divided into a high and low genetic expression group according to the expression level of genes. Survival analysis was considered statistically significant while *P* < 0.05.

The receiver operating characteristic curve (ROC) was used to evaluate the diagnostic efficacy of the indicators and to calculate the area under the curve (AUC) by SPSS 18.0. Next, we analyzed their mRNA expression levels using the GraphPad Prism 7 software combined the corresponding mRNA-seq data of three groups in this case, and also examined the expression of candidate genes in ethnic sub-division of three groups to reflect the potential effects of race on the final results.

## Results

### Data source

Through TCGA database, we obtained complete BC clinical profiles and corresponding RNA-Seq dataset. There were about 7–10% BC patients with *BRCA1/2* mutations and the rest were *BRCA1/2* wild type, obtained from the cBioPortal website; among them, the proportion of *BRCA1* &*BRCA2* mutations was 3% (38/1094, TCGA: Provisional) & 4% (48/1094, TCGA Provisional) (or, 4%,45/1084 & 5%,54/1084, in TCGA: PanCancer Atlas, shown in Fig. S1A). The main mutation type was missense mutation and truncating mutation in *BRCA1* and *BRCA2* genes (Fig. S1B). If only choosing cases with demonstrated mutation in both datasets (TCGA Provisional & TCGA: PanCancer Atlas) and satisfying group criteria, we finally confirmed 42 mutant cases. Demographic data between MUT and WT groups are presented in Table 1.

**Table 1:** Clinical characteristics data for main subjects used in this study.

	MUT group (BRCA1/2-mutant BC tissue)	WT group (randomly selected BC tissue without BRCA1/2 mutations)
N	42	117
Age (initial diagnosis)	57.2 ±13.2	58.3 ±13.2
Race		
White	30/36 (83.3%)	83/109 (76.5%)
African	4/36 (11.1%)	19/109 (17.4%)
Asian	2/36 (5.6%)	7/109 (6.4%)
Stage		
Stage I	4/42 (9.5%)	21/117 (17.9%)
Stage II	32/42 (76.2%)	65/117 (55.6%)
Stage III	6/42 (14.3%)	28/117 (23.9%)
Stage IV	0	3/117 (2.6%)
Immune phenotype		
ER ^−^	18/42 (42.9%)	26/110 (23.6%)
PR ^−^	23/42 (54.8%)	35/109 (32.1%)
HER2		
amplifications	11/39 (28.2%)	15/97 (15.5%)
HER2 ^−^	28/39 (71.8%)	82/97 (84.5%)

### GSEA Enrichment analysis

**Figure 2:**
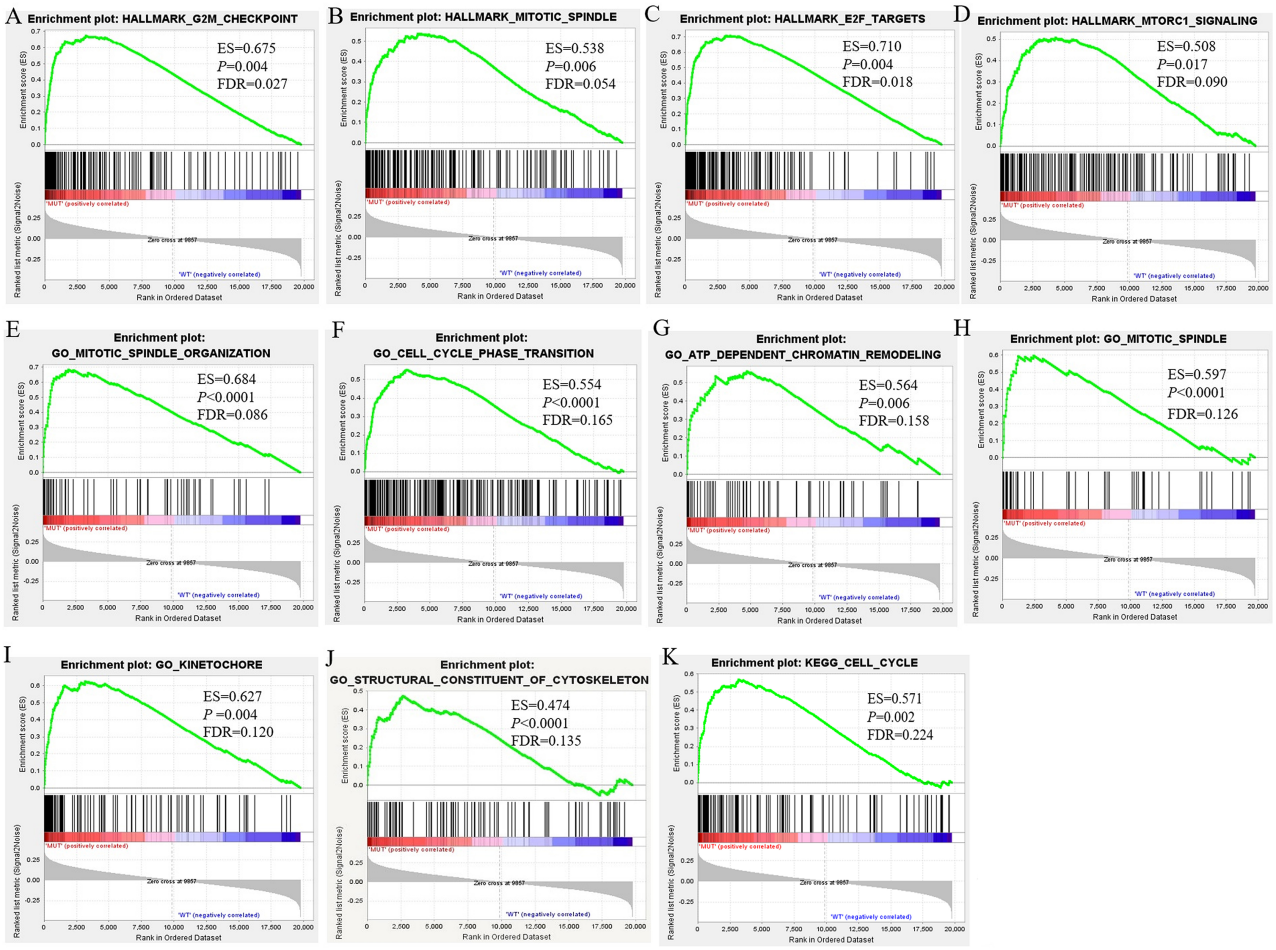
GSEA Enrichment analysis results of BRCA1/2 mutations in breast cancer patients. Note: GSEA Enrichment analysis including H (figure 3A–D), c2 (K) and c5 consisted of biological processes (bp, E–J), cellular component (cc, H–I) and molecular function (mf, J). GSEA, Gene set enrichment analysis.

To investigate the effect of *BRCA1/2* mutations on progression and prognosis of breast cancer, the influences of biological functional annotation sets were analyzed by GSEA method (shown in Fig. 2). The seven, eighty-three or one consensus gene sets from Hallmark collection, c2 KEGG-sub collection or c5 collection, respectively, were significantly enriched in MUT group compared with WT group. Among these enrichment items, gene sets associated with mitotic spindle (e.g., Fig. 2B, Fig. 2E, Fig. 2H), cell cycle (Fig. 2F, Fig. 2K), G2M checkpoint (Fig. 2A) and so on were obviously enriched. Hallmark gene and biological processes pertaining to regulation of transcription involved in G1-S transition, mitotic spindle organization, cell cycle phase transition, ATP dependent chromatin remodeling, cell cycle G1/S transition, negative regulation of cell division, cytoskeleton dependent cytokinesis, cell cycle checkpoint, E2F and MTORC1 signaling, etc., were significantly enriched, suggesting that *BRCA1/2* mutations may contribute to disease progression and affect prognosis mainly by influencing cell proliferation via regulation of cell cycle, cell division and gene replication in breast cancer patients. The GO enrichment analysis of molecular function was significantly enriched in structural constituent of cytoskeleton. Furthermore, the cellular component was enriched for kinetochore, spindle midzone and so on. In the GSEA analysis of KEGG pathways, the *BRCA1/2* mutation group was associated with cell cycle (Fig. 2K).

### Identification of DEGs

**Figure 3:**
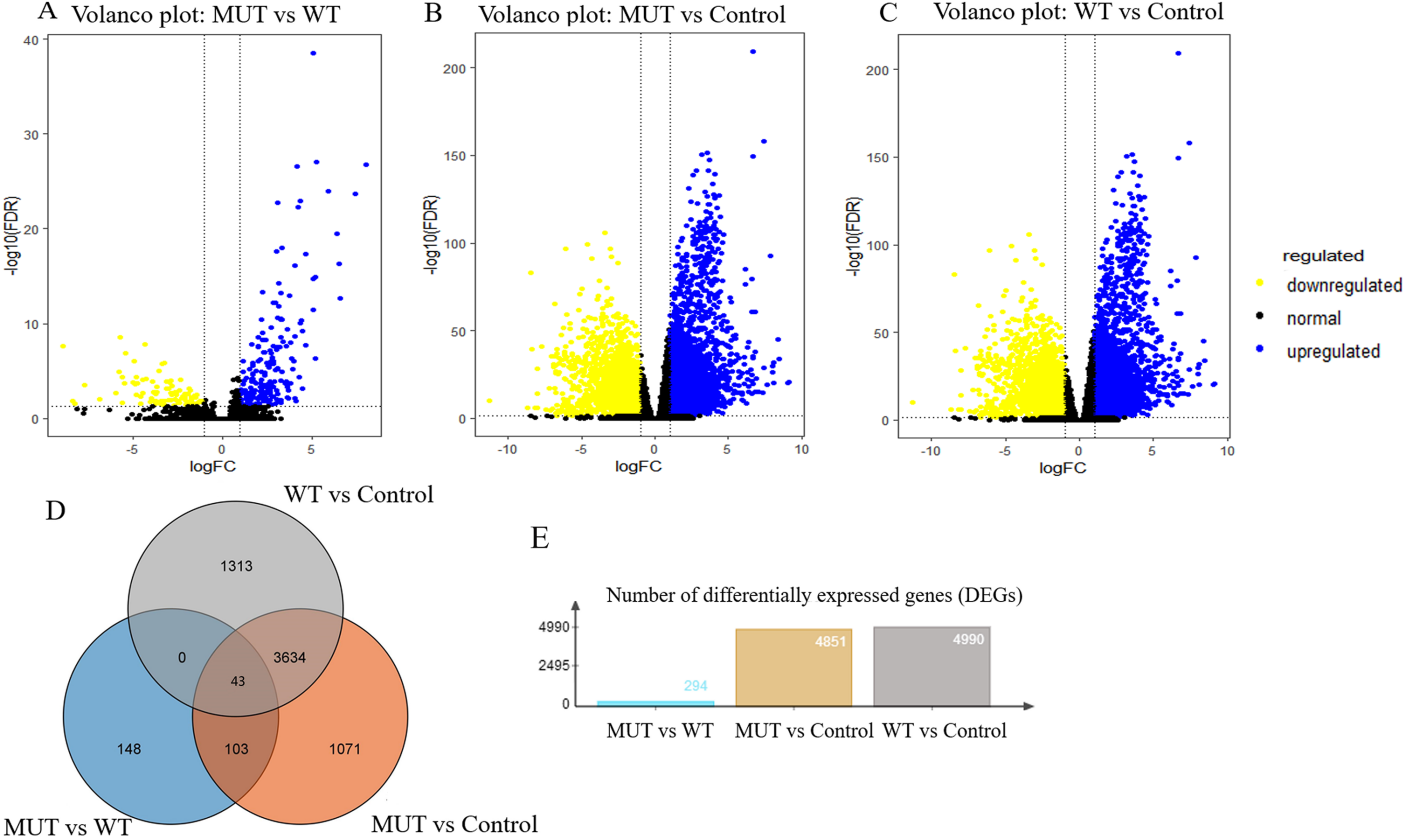
Volcano plot for DEGs and Venn plot of three independent DEGs identifications. Note: As stated earlier, we performed identification of DEGs three times, between *BRCA1/2*-mutant (MUT) group and wild-type (WT) group, MUT and para-carcinoma (Control) group, WT and Control group, respectively. Their volcano plots were shown in figure 3A–C respectively. (D), Venn analysis of above three independent DEGs; (E), the number of differentially expressed genes in three comparisons. Blue: high expression; Yellow: low expression; Black dots: the genes with expression of —log2FC— < 1 or FDR > 0.05. LogFC, log2 fold change; DEGs, differentially expressed genes.

Overall, RNA-Seq datasets from 42 *BRCA1/2* mutation-bearing patients and randomly selected 117 wild-type BC patients were used for DEG screening. A total of 294 DEGs were identified between *BRCA*-mutant and wild-type BC, of which, 199 were upregulated and 95 were downregulated. Furthermore, we performed differentially expressed genes analysis between MUT and control group and identified 4851 differentially expressed genes in MUT group. In addition, comparison of WT (breast cancer) and control group (para-carcinoma tissue) identified 4990 differentially expressed genes in BC patients. The volcano plots of the DEGs were shown in Figs. 3A–3C. More importantly, Venn analysis for three comparisons emphasized a combination of 43 overlapping DEGs (Fig. 3D and Table S1), suggesting the expression of these genes not only significantly changed in breast cancer patients but more obvious significantly changed in *BRCA* 1/2-mutant breast patients. These genes might participate in the specific molecular mechanisms of the carcinogenesis of *BRCA* mutations. The top 10 upregulated and downregulated overlapping DEGs based on fold changes were listed in Table 2.

**Table 2:** The top ten genes with the most obvious expression changes, screened by the identification of DEGs in three comparisons.

Category	Gene symbol	Log FC		
		MUT vs WT	MUT vs control	WT vs control
Top ten upregulated genes in BRCA1/2-mutant breast cancer	CT45A10	4.46	7.66	3.22
	TBX10	3.94	6.63	2.61
	NLRP7	3.26	4.97	1.71
	BARHL2	3.24	5.40	2.15
	C4orf51	2.79	4.63	1.82
	CLLU1OS	2.68	4.15	1.47
	TUBB4A	2.67	4.14	1.43
	NPBWR1	2.32	4.16	1.83
	A2ML1	2.02	4.30	2.27
	TTK	1.00	3.68	2.68
Top ten downregulated genes in BRCA1/2-mutant breast cancer	MYOM2	−3.24	−5.64	−2.42
	CA4	−3.11	−8.48	−5.35
	LGALS12	−2.41	−6.47	−4.04
	SLC4A4	−2.07	−3.48	−1.37
	CAPN11	−1.91	−3.23	−1.36
	HPSE2	−1.90	−5.12	−3.23
	MAOA	−1.89	−4.55	−2.65
	DNAH9	−1.76	−3.21	−1.43
	RELN	−1.75	−4.40	−2.66
	NNAT	−1.55	−4.73	−3.16

### PPI network of DEGs

**Figure 4:**
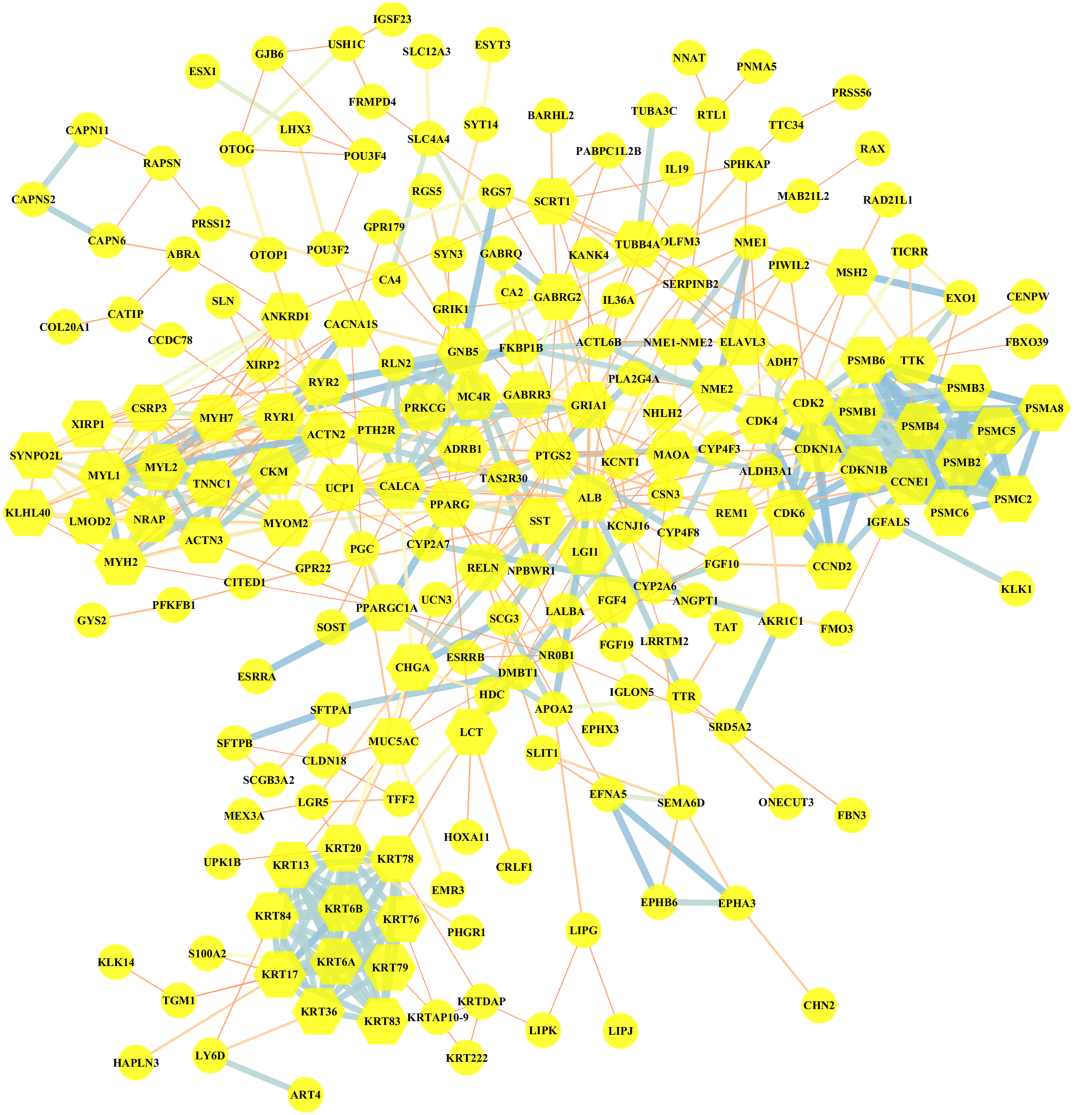
Visualization of the protein–protein interaction (PPI) network of DEGs in MUT vs WT group, by the means of STRING and Cytoscape tool. A total of 294 differentially expressed genes in the comparison of MUT vs. WT were submitted for PPI network construction. A total of 209 nodes and 498 edges were mapped in the PPI network. Yellow, molecules with the node degree > 11.

Altogether, 95 downregulated and 199 upregulated DEGs in MUT vs WT were submitted for further PPI network construction with STRING database and cytoscape software (version 3.7.0), to reflect the specific genetic interaction networks associated with *BRCA1/2* mutations compared with WT group. A total of 209 nodes and 498 edges were mapped in the PPI network (shown in Fig. 4), with an average node degree of 3.34, average local clustering coefficient of 0.372 and a PPI enrichment *P* value <1.0e −16. If all the genes were synthetically evaluated by 12 topological analysis methods from plug-in cytohubba (Chin et al., 2014), we chose top five genes as hub genes for mutant group. These hub genes were serum albumin (*ALB*), *CDKN1A* (cyclin-dependent kinase inhibitor 1), *CCNE1* (G1/S-specific cyclin E1), *MYOM2* (myomesin-2), *KRT20* (keratin 20). Among them, *CCNE1* and *KRT20* were of high expression in *BRCA1/2*-mutant breast cancer, and *ALB* and *MYOM2* were of low expression. Their expression level changes were shown in Table 3.

### Survival analysis and diagnostic efficacy of hub genes

**Figure 5:**
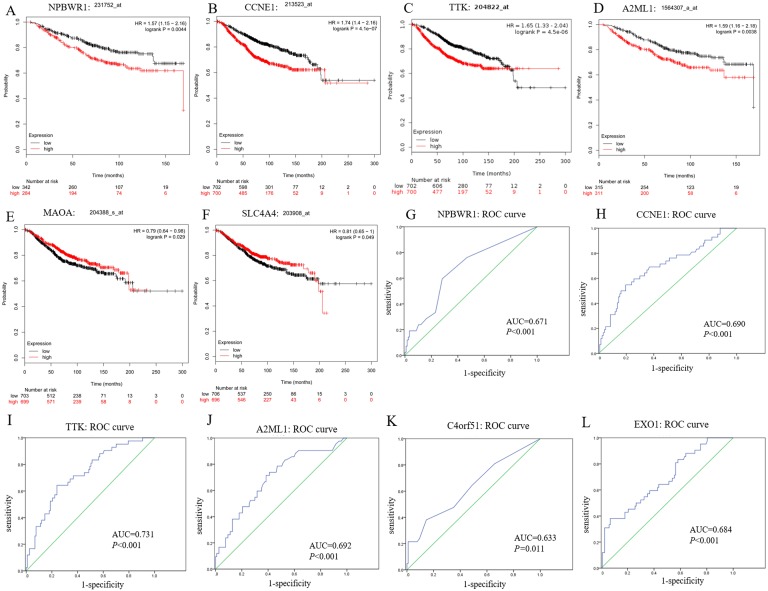
Prognostic and diagnostic significance of identified hub genes for breast cancer. (A–F) illustrate the prognostic value of some hub genes for breast cancer: (A) NPBWR1, (B) CCNE1, (C) TTK, (D) A2ML1, (E) MAOA and (F) SLC4A4. Their prognostic results were obtained from Kaplan–Meier plotter, using the Kaplan–Meier method with a log-tank test, and *P* < 0.05 was considered statistically significant. (G–L) show their ROC curve to reflect the diagnostic values of hub genes to distinguish *BRCA1/2*-mutant breast cancer from wild-type breast cancer. Here, we show (G) NPBWR1, (H) CCNE1, (I) TTK, (J) A2ML1, (K) C4orf51 and (L) EXO1 that were all have good diagnostic efficacy. ROC, the receiver operating characteristic curve.

In this study, we observed *CCNE1*, *NPBWR1* (Neuropeptides B/W receptor 1), *SLC4A4* (Solute Carrier Family 4 member 4), *MAOA* (Monoamine oxidase A), *A2ML1* (Alpha-2-macroglobulin like 1) and *TTK* (dual specificity protein kinase) not only significantly expressed in *BRCA1/2*-mutant breast cancer compared with wild-type BC and normal tissue, but also showed significant prognostic value for breast cancer (shown in Figs. 5A–5F).

Moreover, among upregulated hub genes, we found many genes displayed good diagnostic efficacy for *BRCA1/2*-mutant breast cancer compared to wild-type breast cancer, including *CCNE1*, *NPBWR1*, *A2ML1*, *TTK*, *C4orf51* (Chromosome 4 open reading frame 51) and *EXO1* (Exonuclease 1), with AUC value >0.630 and *P*-value <0.05. Their ROC curves were illustrated in Figs. 5G–5L.

As shown in Fig. S2, although the *NPBWR1*, *A2ML1* and *C4orf51* displayed differential expression, their expression level in breast cancer tissues is still not high (Figs. S2D–S2F), which may be due to their own expression abundance or sensitivity of the detection probe. In view of the fact that the influence of race on genes (*BRCA1/2* and other all genes) is possible but still unknown, we analyzed the relative expression level of hub genes in ethnic subgroups. As shown in Figs. S2A–S2C, we thought that ethnic differences associated with it could be acceptable in general, because the changed expression level of hub genes in the ethnic sub-division of three groups is almost still significant. The lack of data from Asian patients makes it difficult for statistical analysis of Asian ethnic subgroups. Therefore, we believed *CCNE1*, *TTK* and *EXO1* were remarkably overexpressed in BC tissues compared with para-carcinoma tissue, and might be promising to screen BC and further distinguish the high risks of *BRCA1/2* mutations from wild-type BC, while ignoring the potential impact of the genetic background related to the race itself to some extent.

**Table 3:** The expression of some genes with high topological analysis score by cytoscape.

Regulated	Gene symbol	Log FC		
		MUT vs WT	MUT vs Control	WT vs Control
upregulated	CCNE1	1.13	3.28	2.15
	KRT20	7.41	8.03	
downregulated	MYOM2	−3.24	−5.64	−2.42
	ALB	−3.79	−4.42	

## Discussion

The incidence of breast cancer among female worldwide continues to rise, despite the fact that the mortality of cancer has been decreasing due to the development of efficient screening and treatment. The relationship between gene polymorphism and susceptibility to breast cancer, and the influence of multi-gene and multi-signal pathways for the progression of breast cancer have always attracted continuous interests, which will provide ideas for early screening and individualized treatment of breast cancer. Whole genome and exome sequencing including TCGA program have provided novel insights for researchers to explore the complex relationship between genetic molecules and disease, and ultimately advance the precision medicine. From a clinical perspective, precision cancer therapeutics, aims to tailor a treatment strategy to the unique genetic background of each disease, by targeting particular mutants upon exploiting their related mechanistic characterization of the genetic interactions involved in carcinogenesis, tumor progression and metastasis (Tutuncuoglu & Krogan, 2019). In our study, from this thought, we analyzed the difference in genetic expression profiles and interaction networks between mutant and wild-type by selecting transcriptome data from breast cancer patients with *BRCA1/2* mutations.

Mutations in *BRCA1* or *BRCA2* are closely linked to familial breast and ovarian cancers. The *BRCA* genes are tumor suppressor genes that play many critical roles in many tumors, the most important of which is DNA damage repair, especially double-stranded DNA (dsDNA) repair. *BRCA1/2* mutations occur at scattered sites, and occur missense mutations, especially which will influence those situated in exons encoding domains that interact with BRCA1-binding proteins, such as BARD1, BRIP1 and PALB2, which (along with RAD51C, RAD51D and possibly RAP80 and FAM175A, encoding Abraxas) are also breast and/or ovarian cancer susceptibility genes (Foulkes, 2013). Zhao et al. (2017) reported that BRCA1 and its interacting proteins BARD1 functioned in DNA double-strand break repair by influencing RAD51-mediated homologous DNA pairing. In the GSEA enrichment results for *BRCA1/2*-mutant TCGA breast cancer patients, our study also demonstrated *BRCA* genes might be implicated in regulation of tumor cell cycle, through the regulation of cellular component kinetochore and mitotic spindle, and the regulation of cycle-related biological processes including G1/S transition, cell cycle checkpoint and mitotic spindle organization. Through interaction network exploration, we found that *CCNE1* with higher mRNA overexpression in *BRCA1/2*-mutant compared with wild-type BC, might play an important role in cell cycle regulation of *BRCA*-mutant tumors and the tumorgenesis of *BRCA* mutants.

*BRCA* genes mutations, conferring increased risks for breast and high-grade serous cancer of the gynecological tract (fallopian tube, ovary and peritoneum), are closely associated with triple negative breast cancers (TNBC) (Foulkes, 2013; Sanford et al., 2015). Among the classifications of breast cancer, TNBC, manifested as early recurrence and poor survival, does not express estrogen receptor (ER), progesterone receptor (PR), and human epidermal growth factor receptor 2 (HER2). Among patients with TNBC, the incidence of *BRCA1/2* mutation is estimated to range from 11–37% (Sanford et al., 2015; Young et al., 2009). TNBC accounts for about 70% and 16–23% of *BRCA1* and *BRCA2* mutation carriers, respectively (Stevens & Couch, 2013), suggesting that TNBC is also inextricably linked to germline mutation in this breast cancer susceptibility gene. In our study, we also demonstrated that *BRCA1/2*-mutant BC exhibited higher ER receptor and PR receptor negative rates, as well as high HER2 amplification, compared with *BRCA1/2*-wild type BC, by analyzing demographic data from MUT and WT group.

To date, numerous genes have been found to influence the formation and progression of breast cancer, and thus act as diagnostic and therapeutic targets with clinical potentials. Although some genes have been identified and the pathogenic mechanism of *BRCA1/2* genes for breast cancer has partly explained, the closely related genes to *BRCA1/2* in breast cancer remain to be fully elucidated. In our DEGs analysis, the most of the DEGs (a total of 294) obtained from the comparison of *BRCA1/2*-mutant and wild-type breast cancer were demonstrated to be sense; of these, 146 were also differentially expressed between mutant BC and its normal tissue, with upregulated 108 genes and downregulated 38 genes. Furthermore, some genes were further verified by the identification between wild-type BC and normal tissue, which showed that a total of 43 genes had not only significant changes of expression level in BC patients but further more obvious changes in *BRCA1/2*-mutant BC patients. This demonstrated specific expression alternation of genes in our study, including *TTK*, *EXO1*, *TICRR* (TOBPPI interacting checkpoint and replication regulation) and so on, would be closely associated with *BRCA1/2* mutations, providing a clue for further understanding the certain characteristic of *BRCA1/2*-mutant BC (for example, increased risk of distant metastasis and more aggressive nature) (Wang et al., 2018) and clarifying its pathogenesis.

More interestingly, some candidate genes displayed potential therapeutic and diagnostic value, especially for *BRCA1/2*-mutant breast cancer. For example, upregulated *CCNE1*, *TTK* and *EXO1* displayed good diagnostic efficacy for screening breast cancer and *BRCA1/2*-mutant breast cancer. We noted that the *BRCA1/2* mutations rate of the White is a bit higher in WT group than that in MUT group, which might influence the reliability of our results and become an existing problem. TCGA data is mainly derived from White patients, so it could be difficult to achieve strict inter-group ethnic balance. However, in view of possible ethnical risks with *BRCA1/2* (De Bruin et al., 2012), by analyzing the relative expression level of hub genes in ethnic subgroups, we thought that the potential impact of ethnic differences could be acceptable in general. Of course, it was important to note that the differences in gene expression of some genes in the Asian subgroup are sometimes insignificant, which was be due to the lack of Asian patients’ samples in TCGA data itself. Through Kaplan–Meier approach, some differentially expressed genes with good PPI network scores were evaluated their prognosis information. Herein, we only analyzed the prognostic value of these genes for the whole breast cancer due to lack of adequate *BRCA1/2*-mutant cases for accurate survival analysis, which was an undeniable limitation in our study. Using the survival analysis, we found that *CCNE1*, *NPBWR1, A2ML1* and *TTK* might act critical functions in the oncogenesis and progression of *BRCA1/2*-mutant breast cancer, reflected by their diagnostic efficacy for *BRCA1/2* mutations and prognostic value as well.

*EXO1*, a DNA mismatch repair gene, its polymorphisms have been reported to play a critical role in the development of many tumors (Shi et al., 2017; Zhang et al., 2016). Also, due to its role in DNA replication repair and homology-directed repair, the relationships of EXO1 *and BRCA1/2* mutations and its underlying mechanism have become an important focus to be studied (Lemacon et al., 2017). A recent study found that the *EXO1* expression level was elevated in hepatocellular carcinoma patients and its overexpression was correlated with larger tumor size, increased lymph node metastasis, and thus proving its potential therapeutic value for hepatocellular carcinoma as a promising prognostic marker (Dai et al., 2018). Our findings were validated by another study to some extent, and they found DEPDC1, EXO1, RRM2 and some proteins had enhanced expression in the ductal carcinoma in situ and invasive ductal carcinoma (Kretschmer et al., 2011). In a word, the functions of EXO1 still require further research to fully illuminate its role in the progression of BC and the carcinogenesis of *BRCA1/2* mutations.

The protein encoded by *CCNE1* is G1-S specific cyclin that plays an important role in regulating the transition of G1 to S cell cycle phase by binding to and activating the expression of cyclin-dependent protein kinase 2 (Cdk2) (Bendris & Blanchard, 2015). CCNE1 also has a direct role in triggering DNA replication and maintaining genomic stability. Amplification or upregulated expression of *CCNE1* is associated with poor prognosis in some tumors such as breast or ovarian cancer (Karst et al., 2014; Zhao et al., 2019). In our analysis, *CCNE1* was significantly upregulated in *BRCA1/2*-mutant BC, compared with wild-type BC, suggesting that *BRCA1/2* genes could regulate cell cycle in tumors via CCNE1 reflected by the fact that cell cycle phase transition, especially cell cycle G1/S transition were significantly enriched in mutant breast cancer from the GSEA results.

NPBW1 or GPR7 (namely Neuropeptides B/W receptor 1), a protein encoded by the *NPBWR1* gene, can mainly regulate physiological responses related to the nervous system, including stress response and pain response (Nagata-Kuroiwa et al., 2011). However, the specific mechanism of NPBW1 in tumorigenesis has not been studied and confirmed. Cottrell S et al. reported that methylation of *GPR7* was significantly associated with prostate cancer prognosis, and would result in more accurate prediction for prostate cancer recurrence in clinical practice (Cottrell et al., 2007). A2ML1 is a broad protease inhibitor from the alpha-macroglobulin superfamily, with a unique mechanism where A2ML1 undergoes a conformational change following its cleavage by a protease and thereby traps the protease to prevent proteases from binding to their substrates (Galliano et al., 2006; Vissers et al., 2015). The clinical significance of A2ML1 has been demonstrated in paraneoplastic pemphigus (PNP), an autoimmune bullous disease accompanied by a variety of benign or malignant tumors including non-Hodgkin lymphoma (Mimouni et al., 2002). A2ML1 could serve as a useful diagnostic biomarker for PNP (Ohzono et al., 2015). Recently, a bioinformatics study pointed out the new use of A2ML1 as a diagnostic target for lung cancer (Zhang et al., 2018). In our study, we found that *NPBWR1* and *A2ML1* have certain prognostic or diagnostic significance for breast cancer, and we thought that the two molecules also deserve further investigated, though their expression level in breast cancer tissues is not high enough overall.

At present, many studies have found that high expression of dual specificity protein kinase (TTK), encoded by the *TTK* gene, is associated with the oncogenesis, progression and treatment resistance of breast cancer (especially TNBC) (Riggs et al., 2017). It was reported that TTK could regulate the growth and epithelial-to-mesenchymal transition of TNBC cells through TGF- β and KLF5 signal pathways, thereby affecting the invasion and metastasis of tumors (King et al., 2018). More future research about its mechanisms in TNBC will provide a theoretical basis for TTK inhibitor-targeted therapy in the field of breast cancer and TNBC. In the present study, the upregulated expression and corresponding diagnostic value of *TTK* in *BRCA1/2*-mutant breast cancer suggested that the role of TTK in this type of breast cancer is equally noteworthy.

Here, by many bioinformatic analyses, we identified some important molecules affected by *BRCA* genes mutations. Survival and diagnosis analysis, and the validation of genes in the ethnic sub-groups implied their potentials as reliable prognostic or diagnostic indicators and as possible therapeutic targets. In this article, from a new perspective, we identified some novel DEGs, including *CCNE1*, *NPBWR1*, *A2ML1*, *EXO1* and *TTK*, might play critical functions in the oncogenesis and progression of *BRCA1/2*-mutant BC, which was not been previously interpreted from a similar idea. Of course, it is necessary to indicate that *BRCA1/2*-mutant patients in this study are derived from the gene mutations detection of TCGA cancer patients, who are not distinguished between somatic mutations and germline mutations (not necessarily as hereditary breast cancer patients). Moreover, in order to select more credible key candidate genes, the selection of the hub genes was validated in other comparisons of mutant and normal tissues, and of wild-type and normal tissues. In a word, our study will help explain the underlying mechanisms of BRCA in carcinogenesis, identify novel diagnostic indicators for breast cancer and *BRCA1/2*-mutant breast cancer, and provide new targets and strategies for personalized therapy.

## Conclusion

By bioinformatic analyses including GSEA enrichment analysis (GO and KEGG), differentially expressed genes identification, PPI network, survival and diagnostic value analysis, we identified *CCNE1*, *TTK* and *EXO1* might act as the potential diagnostic indicators for screening BC and *BRCA1/2*-mutant BC. Our results revealed that cell cycle regulation, cell division and proliferation may play crucial roles in *BRCA1/2* mutation BC. A total of 43 overlapping DEGs might play critical functions in the oncogenesis and progression of *BRCA1/2*-mutant BC, reflected by their specifically changed expression levels in *BRCA* mutant carriers compared with wild-type BC. Also, *CCNE1* and *TTK* might serve as prognostic biomarkers for BC. However, further validation by molecular biological experiments are required to confirm our investigation. Additional findings obtained in our study (other changed genetic molecules) are also worthy further research.

##  Supplemental Information

10.7717/peerj.8403/supp-1 Supplemental Information 1 More raw measurements in our study, including grouping information, sample ID from TCGA dataset and enrichment analysis

10.7717/peerj.8403/supp-2 Table S1 Differentially expressed genes in three comparisons The overlapping genes in three independent DEGs identifications by Venn analysis.

10.7717/peerj.8403/supp-3 Supplemental Information 2 Figure S1 and S2

## Abbreviations

BRCA1/2: Breast cancer susceptibility gene 1/2
BC: breast cancer
TNBC: Triple-negative breast cancer
TCGA: The Cancer Genome Atlas
DEGs: Differentially expressed genes
PPI: Protein–protein interaction
GSEA: Gene set enrichment analysis
KEGG: Kyoto Encyclopedia of Genes and Genomes
CNV: copy number variation
GO: Gene Ontology
BP: biological process
FDR: false discovery rate
FC: fold change
ROC: The receiver operating characteristic curve
AUC: the area under the curve
ER: Estrogen receptor
PR: Progesterone receptor
HER2: Human epidermal growth factor receptor 2
OS: Overall survival
mTORC1: mechanistic target of rapamycin complex 1
ALB: serum albumin
CDKN1A: Cyclin-dependent kinase inhibitor 1
CCNE1: cyclin E1
MYOM2: myomesin-2
KRT20: keratin 20
NPBWR1: Neuropeptides B/W receptor 1
SLC4A4: Solute Carrier Family 4 member 4
MAOA: Monoamine oxidase A
NLRP7: NLR family pyrin domain containing 7
C4orf51: Chromosome 4 open reading frame 51
A2ML1: Alpha-2-macroglobulin like 1
TTK: TTK protein kinase or dual specificity protein kinase

## Additional Information and Declarations

### Data Availability

The following information was supplied regarding data availability: Raw measurements are available in the Supplemental Files.

## References

[ref-1] Armstrong N, Ryder S, Forbes C, Ross J, Quek RGW (2019). A systematic review of the international prevalence of BRCA mutation in breast cancer. Clinical Epidemiology.

[ref-2] Atchley DPAC, Lopez A, Valero V, Amos CI, Gonzalez-Angulo AM, Hortobagyi GN, Arun BK (2008). Clinical and pathologic characteristics of patients with BRCA-positive and BRCA-negative breast cancer. Journal of Clinical Oncology.

[ref-3] Barber LJSS, Chen L, Campbell J, Kozarewa I, Fenwick K, Assiotis I, Rodrigues DN, Reis Filho JS, Moreno V, Mateo J, Molife LR, De Bono J, Kaye S, Lord CJ, Ashworth A (2013). Secondary mutations in BRCA2 associated with clinical resistance to a PARP inhibitor. Journal of Pathology.

[ref-4] Bendris NLB, Blanchard JM (2015). Cell cycle, cytoskeleton dynamics and beyond: the many functions of cyclins and CDK inhibitors. Cell Cycle.

[ref-5] Bernstein-Molho RB-KI, Ludman MD, Reznik G, Feldman HB, Samra NN, Eilat A, Peretz T, Peretz LP, Shapira T, Magal N, Kalis ML, Yerushalmi R, Vinkler C, Liberman S, Basel-Salmon L, Shohat M, Levy-Lahad E, Friedman E, Bazak L, Goldberg Y (2019). The yield of full BRCA1/2 genotyping in Israeli Arab high-risk breast/ovarian cancer patients. Breast Cancer Research and Treatment.

[ref-6] Chin CHCS, Wu HH, Ho CW, Ko MT, Lin CY (2014). cytoHubba: identifying hub objects and sub-networks from complex interactome. BMC Systems Biology.

[ref-7] Cottrell SJK, Kristiansen G, Eltze E, Semjonow A, Ittmann M, Hartmann A, Stamey T, Haefliger C, Weiss G (2007). Discovery and validation of 3 novel DNA methylation markers of prostate cancer prognosis. Journal d Urologie.

[ref-8] Dai Y, Tang Z, Yang Z, Zhang L, Deng Q, Zhang X, Yu Y, Liu X, Zhu J (2018). EXO1 overexpression is associated with poor prognosis of hepatocellular carcinoma patients. Cell Cycle.

[ref-9] De Bruin MA, Kwong A, Goldstein BA, Lipson JA, Ikeda DM, McPherson L, Sharma B, Kardashian A, Schackmann E, Kingham KE, Mills MA, West DW, Ford JM, Kurian AW (2012). Breast cancer risk factors differ between Asian and white women with BRCA1/2 mutations. Familial Cancer.

[ref-10] Foulkes WDSA (2013). In Brief: BRCA1 and BRCA2. Journal of Pathology.

[ref-11] Galliano M-F, Toulza E, Gallinaro H, Jonca N, Ishida-Yamamoto A, Serre G, Guerrin M (2006). A novel protease inhibitor of the alpha2-macroglobulin family expressed in the human epidermis. Journal of Biological Chemistry.

[ref-12] Gao JAB, Dogrusoz U, Dresdner G, Gross B, Sumer SO, Sun Y, Jacobsen A, Sinha R, Larsson E, Cerami E, Sander C, Schultz N (2013). Integrative analysis of complex cancer genomics and clinical profiles using the cBioPortal. Science Signaling.

[ref-13] Gourley C (2019). Population BRCA sequencing; time to move to the next phase?. An International Journal of Obstetrics & Gynaecology.

[ref-14] Gyorffy BLA, Eklund AC, Denkert C, Budczies J, Li Q, Szallasi Z (2010). An online survival analysis tool to rapidly assess the effect of 22, 277 genes on breast cancer prognosis using microarray data of 1809 patients. Breast Cancer Research and Treatment.

[ref-15] Huang RLX, Li Q (2017). Identification of key pathways and genes in TP53 mutation acute myeloid leukemia: evidence from bioinformatics analysis. OncoTargets and Therapy.

[ref-16] Karst AMJP, Vena N, Ligon AH, Liu JF, Hirsch MS, Etemadmoghadam D, Bowtell DD, Drapkin R (2014). Cyclin E1 deregulation occurs early in secretory cell transformation to promote formation of fallopian tube-derived high-grade serous ovarian cancers. Cancer Research.

[ref-17] King JLZB, Li Y, Li KP, Ni JJ, Saavedra HI, Dong JT (2018). TTK promotes mesenchymal signaling via multiple mechanisms in triple negative breast cancer. Oncogenesis.

[ref-18] Kretschmer C, Sterner-Kock A, Siedentopf F, Schoenegg W, Schlag PM, Kemmner W (2011). Identification of early molecular markers for breast cancer. Molecular Cancer.

[ref-19] Kuchenbaecker KBHJ, Barnes DR, Phillips KA, Mooij TM, Roos-Blom MJ, Jervis S, Van Leeuwen FE, Milne RL, Andrieu N, Goldgar DE, Terry MB, Rookus MA, Easton DF, Antoniou AC, McGuffog L, Evans DG, Barrowdale D, Frost D, Adlard J, Ong KR, Izatt L, Tischkowitz M, Eeles R, Davidson R, Hodgson S, Ellis S, Nogues C, Lasset C, Stoppa-Lyonnet D, Fricker JP, Faivre L, Berthet P, Hooning MJ, Van der Kolk LE, Kets CM, Adank MA, John EM, Chung WK, Andrulis IL, Southey M, Daly MB, Buys SS, Osorio A, Engel C, Kast K, Schmutzler RK, Caldes T, Jakubowska A, Simard J, Friedlander ML, McLachlan SA, Machackova E, Foretova L, Tan YY, Singer CF, Olah E, Gerdes AM, Arver B, Olsson H, BRCA1 and BRCA2 Cohort Consortium (2017). Risks of breast, ovarian, and contralateral breast Cancer for BRCA1 and BRCA2 mutation carriers. Journal of the American Medical Association.

[ref-20] Lemacon D, Jackson J, Quinet A, Brickner JR, Li S, Yazinski S, You Z, Ira G, Zou L, Mosammaparast N, Vindigni A (2017). MRE11 and EXO1 nucleases degrade reversed forks and elicit MUS81-dependent fork rescue in BRCA2-deficient cells. Nature Communications.

[ref-21] Liberzon ABC, Thorvaldsdottir H, Ghandi M, Mesirov JP, Tamayo P (2015). The molecular signatures database (MSigDB) hallmark gene set collection. Cell Systems.

[ref-22] Macedo GSAB, Ashton-Prolla P (2019). Reviewing the characteristics of BRCA and PALB2-related cancers in the precision medicine era. Genetics and Molecular Biology.

[ref-23] Mimouni D, Anhalt GJ, Lazarova Z, Aho S, Kazerounian S, Kouba DJ, Mascaro Jr JM, Nousari HC (2002). Paraneoplastic pemphigus in children and adolescents. British Journal of Dermatology.

[ref-24] Nagata-Kuroiwa RFN, Hara J, Hondo M, Ishii M, Abe T, Mieda M, Tsujino N, Motoike T, Yanagawa Y, Kuwaki T, Yamamoto M, Yanagisawa M, Sakurai T (2011). Critical role of neuropeptides B/W receptor 1 signaling in social behavior and fear memory. PLOS ONE.

[ref-25] Ohzono A, Sogame R, Li X, Teye K, Tsuchisaka A, Numata S, Koga H, Kawakami T, Tsuruta D, Ishii N, Hashimoto T (2015). Clinical and immunological findings in 104 cases of paraneoplastic pemphigus. British Journal of Dermatology.

[ref-26] Riggs JRNM, Elsner J, Erdman P, Cashion D, Robinson D, Harris R, Huang D, Tehrani L, Deyanat-Yazdi G, Narla RK, Peng X, Tran T, Barnes L, Miller T, Katz J, Tang Y, Chen M, Moghaddam MF, Bahmanyar S, Pagarigan B, Delker S, LeBrun L, Chamberlain PP, Calabrese A, Canan SS, Leftheris K, Zhu D, Boylan JF (2017). The discovery of a dual TTK protein kinase/CDC2-like kinase (CLK2) inhibitor for the treatment of triple negative breast cancer initiated from a phenotypic screen. Journal of Medicinal Chemistry.

[ref-27] Sanford RASJ, Gutierrez-Barrera AM, Profato J, Woodson A, Litton JK, Bedrosian I, Albarracin CT, Valero V, Arun B (2015). High incidence of germline BRCA mutation in patients with ER low positive/PR low positive/HER-2 neu negative tumors. Cancer.

[ref-28] Santana Dos Santos E, Lallemand FBL, Stoppa-Lyonnet D, Brown M, Caputo SM, Rouleau E (2018). Non-coding variants in BRCA1 and BRCA2 genes: potential impact on breast and ovarian cancer predisposition. Cancer.

[ref-29] Shi T, Jiang R, Wang P, Xu Y, Yin S, Cheng X, Zang R (2017). Significant association of the EXO1 rs851797 polymorphism with clinical outcome of ovarian cancer. OncoTargets and Therapy.

[ref-30] Shimada SYR, Nakashima E, Kitagawa D, Gomi N, Horii R, Takeuchi S, Ashihara Y, Kita M, Akiyama F, Ohno S, Saito M, Arai M (2019). Five screening-detected breast cancer cases in initially disease-free BRCA1 or BRCA2 mutation carriers. Breast Cancer.

[ref-31] Stevens KNVC, Couch FJ (2013). Genetic susceptibility to triple-negative breast cancer. Cancer Research.

[ref-32] Tutuncuoglu B, Krogan NJ (2019). Mapping genetic interactions in cancer: a road to rational combination therapies. Genome Medicine.

[ref-33] Vissers LE, Bonetti M, Overman JP, Nillesen WM, Frints SGM, Ligt JD, Zampino G, Justino A, Machado JC, Schepens M, Brunner HG, Veltman JA, Scheffer H, Gros P, Costa JL, Tartaglia M, Burgt IVD, Yntema HG, Hertog JD (2015). Heterozygous germline mutations in A2ML1 are associated with a disorder clinically related to Noonan syndrome. European Journal of Human Genetics.

[ref-34] Wang YA, Jian JW, Hung CF, Peng HP, Yang CF, Cheng HS, Yang AS (2018). Germline breast cancer susceptibility gene mutations and breast cancer outcomes. BMC Cancer.

[ref-35] Young SRPR, Donenberg T, Shapiro C, Hammond LS, Miller J, Brooks KA, Cohen S, Tenenholz B, Desai D, Zandvakili I, Royer R, Li S, Narod SA (2009). The prevalence of BRCA1 mutations among young women with triple-negative breast cancer. BMC Cancer.

[ref-36] Zhai QLH, Sun L, Yuan Y, Wang X (2019). Identification of differentially expressed genes between triple and non-triple-negative breast cancer using bioinformatics analysis. Breast Cancer.

[ref-37] Zhang M, Zhao D, Yan C, Zhang L, Liang C (2016). Associations between nine polymorphisms in EXO1 and cancer susceptibility: a systematic review and meta-analysis of 39 case-control studies. Scientific Reports.

[ref-38] Zhang W, Cui Q, Qu W, Ding X, Jiang D, Liu H (2018). TRIM58/cg26157385 methylation is associated with eight prognostic genes in lung squamous cell carcinoma. Oncology Reports.

[ref-39] Zhao WSJ, Liang F, Chen X, Maranon DG, Jian Ma C, Kwon Y, Rao T, Wang W, Sheng C, Song X, Deng Y, Jimenez-Sainz J, Lu L, Jensen RB, Xiong Y, Kupfer GM, Wiese C, Greene EC, Sung P (2017). BRCA1-BARD1 promotes RAD51-mediated homologous DNA pairing. Nature.

[ref-40] Zhao ZMYS, Hutchinson KE, Li SM, Yuan YC, Noorbakhsh J, Liu Z, Warden C, Johnson RM, Wu X, Chuang JH, Yuan Y (2019). CCNE1 amplification is associated with poor prognosis in patients with triple negative breast cancer. BMC Cancer.

