# Supplementary material for: Differentially expressed genes and key molecules of BRCA1/2-mutant breast cancer: evidence from bioinformatics analyses
Source: PeerJ. 2020 Jan 21;8:e8403. doi: 10.7717/peerj.8403 (PMC6979404; doi:10.7717/peerj.8403)
Supplement: Supplemental Information 2 [file peerj-08-8403-s003.docx]

**Supplement** **information-figure S1**


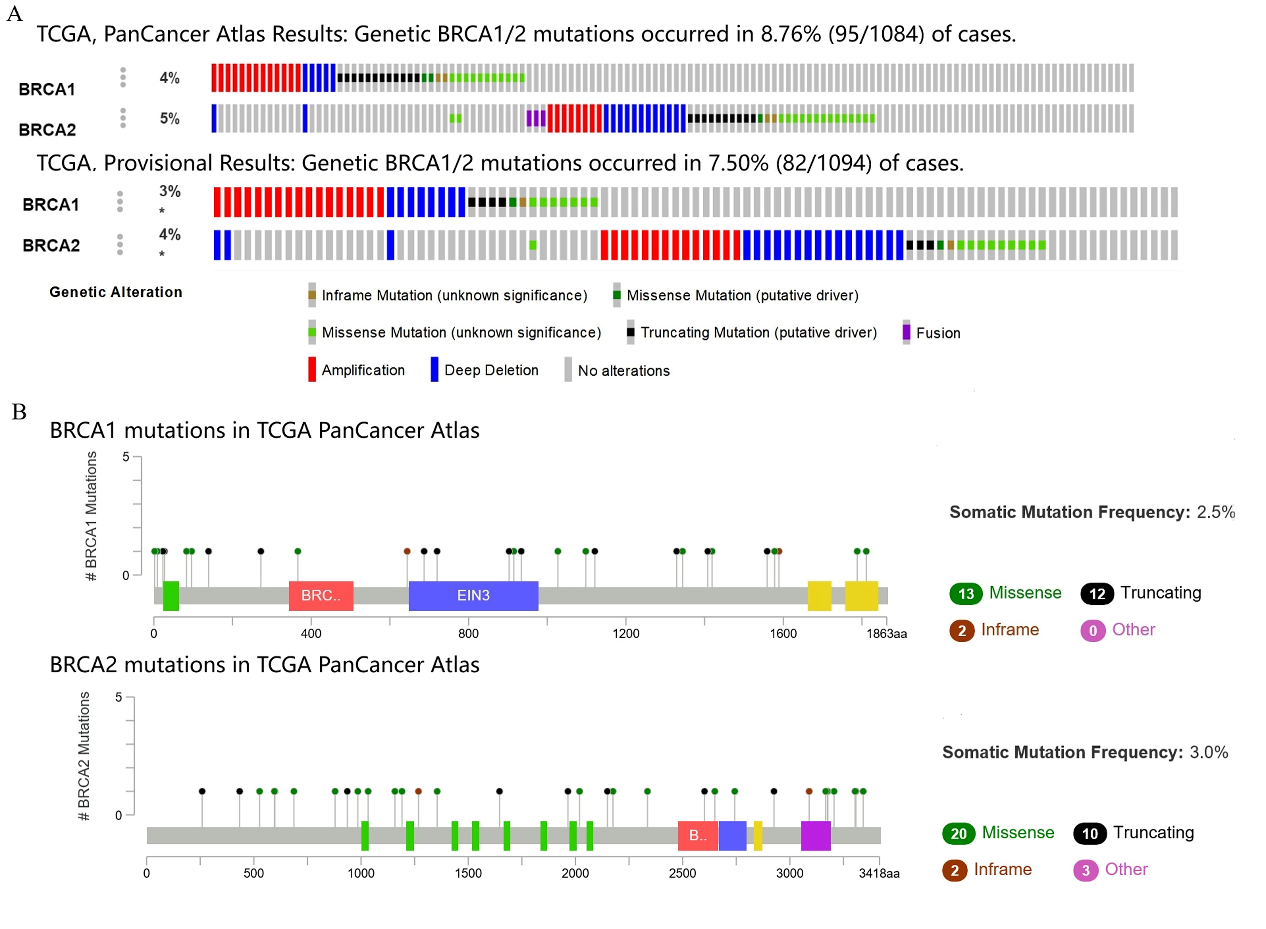


Figure S1 *BRCA1/2* mutations in TCGA database using cBioPortal.

Note: A, *BRCA1* and *BRCA2* mutations including genetic alteration and genetic copy number alteration, in TCGA PanCancer Atlas and TCGA Provisional data set. B, *BRCA1/2* Mutation type in TCGA PanCancer Atlas dataset, mainly includes missense mutation and truncating mutation, similar to another TCGA dataset.

**Supplement information-table S1**

Table S1 The overlapping differentially expressed genes (DEGs) in three comparisons.

See file (TableS1.xls). The table showed the overlapping DEGs in three comparisons, or in MUT vs WT and MUT vs control, by Venn analysis, respectively. Number of the tableS1 is log2 fold change (logFC) values of each gene in the set, calculated as mean gene expression in the experimental group divided by mean gene expression in the corresponding control group and then take the log base 2. LogFC, log fold change.

**Supplement information-figure S2**


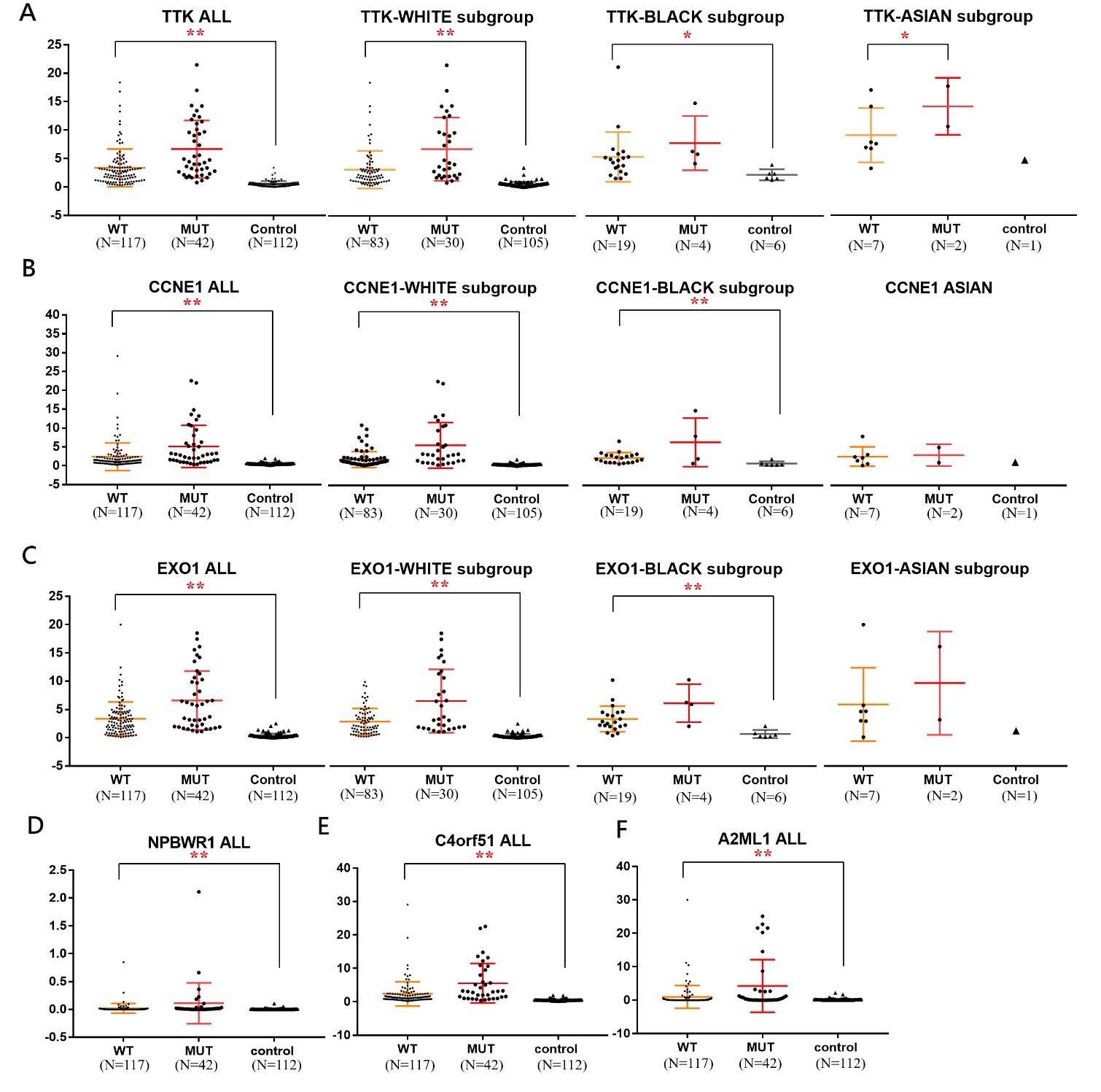


Figure S2 The relative expression level of hub genes in three groups and corresponding ethnic subgroups.

Note: The figure showed the relative expression level of hub genes in three groups (MUT, WT and control group) and corresponding ethnic subgroups, to reflect the potential effect of race on hub genes with good diagnostic value for breast cancer and *BRCA1/2*-mutant breast cancer: *TTK* (figure-S2A), *CCNE1* (figure-S2B) and *EXO1* (figure-S2C), respectively. We noted that although the *NPBWR1* (figure S2D), *C4orf51* (figure S2E) and *A2ML1* (figure S2F) displayed significant differential expression, their expression level in breast cancer tissues is still not high. MUT, *BRCA1/2*-mutant breast cancer; WT, *BRCA1/2* wild-type breast cancer; Control, para carcinoma tissues in TCGA breast cancer dataset. **, *P*<0.01; *, *P*<0.05.
